# Mechanisms of astrocyte aging in reactivity and disease

**DOI:** 10.1186/s13024-025-00810-7

**Published:** 2025-02-21

**Authors:** Holly K. Gildea, Shane A. Liddelow

**Affiliations:** 1 Institute for Translational Neuroscience, NYU Grossman School of Medicine, New York, USA; 2 Department of Neuroscience, NYU Grossman School of Medicine, New York, USA; 3 Department of Ophthalmology, NYU Grossman School of Medicine, New York, USA; 4 Parekh Center for Interdisciplinary Neurology, NYU Grossman School of Medicine, New York, USA; 5 Optimal Aging Institute, NYU Grossman School of Medicine, New York, USA

**Keywords:** Aging, Astrocyte, Glia, Astrocyte reactivity, Neurodegenerative disease, Lipid droplets, Mitochondria, Proteostasis, Senescence, Inflammaging

## Abstract

Normal aging alters brain functions and phenotypes. However, it is not well understood how astrocytes are impacted by aging, nor how they contribute to neuronal dysfunction and disease risk as organisms age. Here, we examine the transcriptional, cell biology, and functional differences in astrocytes across normal aging. Astrocytes at baseline are heterogenous, responsive to their environments, and critical regulators of brain microenvironments and neuronal function. With increasing age, astrocytes adopt different immune-related and senescence-associated states, which relate to organelle dysfunction and loss of homeostasis maintenance, both cell autonomously and non-cell autonomously. These perturbed states are increasingly associated with age-related dysfunction and the onset of neurodegeneration, suggesting that astrocyte aging is a compelling target for future manipulation in the prevention of disease.

## Background

Aging in humans is associated with increased risk of neurological disease, cognitive impairment, and worsened outcomes following infection or trauma. The age-related onset of neuropathological conditions and/or abnormal responses to pathology suggest an important role for central nervous system (CNS) aging in the context of the whole organism. Understanding how normal aging changes the brain will provide insights into the pathogenesis of degenerative diseases. Despite decades of neuron-centric study surrounding aging and neurodegeneration, recent work suggests that non-neuronal glial cells are the most transcriptionally altered in the aging brain [1]. Among these glia, astrocytes are best known for their metabolic, structural, and activity-regulating functions on behalf of neurons, though recent work has expanded understanding of astrocyte roles into disease, cognition, and beyond [2].

Normal aging leads to a decline in homeostasis maintenance across tissues, particularly in regulation of organelle functions and response to damage (for review see [3]). Across organ systems, key mitochondrial, proteostatic, and damage handling pathways decline during aging [for review see 4]. In the CNS, many of these regulatory functions are allocated to astrocytes under homeostatic conditions to enable efficient neuronal functioning. Astrocytes are key regulators of metabolism and energy generation that also sense and handle damage downstream of these and other cellular processes. Astrocytes are required for maintenance and regulation of synapse stability and neuronal activity, which become perturbed in advanced aging [5] (for review see [6, 7]). Neurons also offload damaged species like dysfunctional organelles and reactive oxygen species-affected lipids to astrocytes for degradation [8, 9]. Understanding how astrocytes regulate these processes under homeostatic conditions and how normal functions decline during aging is crucial to our analysis of brain aging phenotypes and degeneration.

Astrocytes are perhaps best described for their roles in responding to insults, such as disease, infection/inflammation, neuronal trauma, and perturbations of organismal metabolism [10–14]. In addition to the many functions performed by these cells under homeostasis, astrocytes under stress react to unique circumstances by enacting unique responses [13, 15, 16]. These stress-responsive astrocytes, termed “reactive astrocytes,” can lose homeostatic capabilities and/or gain additional functions such as proliferation and scar formation, neurotoxicity, or immune cell regulation, among others (for review see [2, 17]). The context dependent and multifaceted nature of astrocyte reactivity suggests that states of astrocytes during normal aging are likely reliant on extrinsic cues that accumulate across the lifespan. For example, aging is associated with increased inflammation and infection, as well as senescence and metabolic disease [for review see 4]. How astrocytes synthesize these cues during aging and alter their baseline states is largely unknown.

Specific changes in aged astrocytes, both intrinsic and related to their long-term cell–cell interactions as organisms age, are poorly understood and have been difficult to interrogate with high fidelity. New and developing analytical tools such as single cell sequencing and multi-omic characterization strategies have begun to describe aged astrocytes, but more work is needed to fully understand the functional consequences of these alterations and how changes occur in different contexts and disease conditions. Improved functional characterization of aged astrocytes will likely provide insight into aging-related disease mechanisms and propose avenues to address aging brain phenotypes moving forward.

### Tools and strategies for evaluating aged astrocyte transcriptomes

Modern transcriptome technologies have expanded our characterization of aged astrocytes; however, several tool limitations and facets of aging biology still limit our knowledge. Lowly abundant transcripts change in phenotypically relevant ways across the lifespan; for example, lowly-expressed stress response factors and chaperones have major roles in aging biology [18] (for review see also [4, 19]). Small changes on the individual transcript level can also represent a hard-to-detect aging-related whole-genome transcription length bias [20]. Interrogation of such transcripts requires excellent sequencing depth for accurate detection of age-associated changes. In old animals, debris contamination is exacerbated by increased myelination and decreased tissue quality, especially in post-mortem human tissue, requiring additional physical and computational clean-up. Risk of promoter leakage also increases with cellular age, requiring rigorous purification and analysis strategies to limit contamination and focus on real signal particularly for long-lived cells of the CNS [21]. Important subtypes (regional or permanent identity) and substates (reactive to stimuli, often acute) of astrocytes may be under-represented in bulk or whole-brain samples, as astrocytes are difficult to capture relative to neurons and other cells [22], and investigators may prioritize maintaining key sequencing depth over understanding spatial context and cellular resolution in aging studies.

**Fig. 1:**
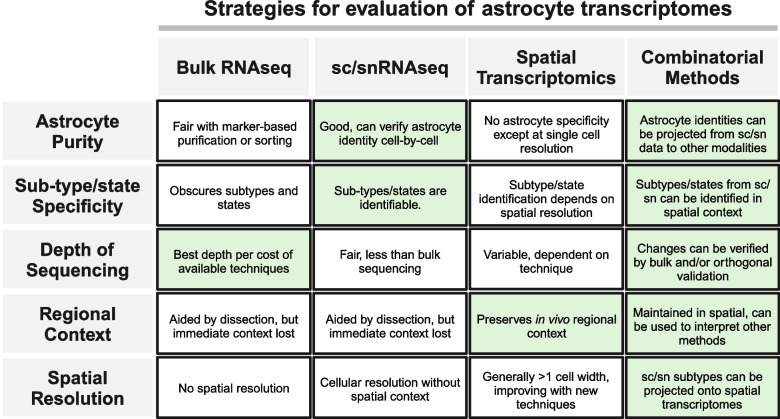
Strategies for characterizing astrocyte transcriptomics (columns) described across efficacy in several modalities (rows). Green shaded backgrounds indicate stronger performance in each area

To overcome problems in collection and interpretation of aged astrocyte sequencing, integrative methods that evaluate astrocyte aging across regions, contexts, and analysis methods are likely to be most useful (Fig. 1). In young astrocytes, for example, combining transcriptomic and proteomic data streams allowed alignment of region-specific subtypes across the CNS [23]. Improved spatial transcriptomics methods now allow evaluation of region-specific changes at single cell resolution, and several groups have identified astrocyte signatures in single cell/nucleus RNA sequencing (sc/snRNAseq) that reliably cross-reference with regional subtypes in spatial transcriptomics [24, 25]. Newer strategies allow the incorporation of non-transcriptional methods such as spatial proteomics [for review see 26], metabolomics [27], lipidomics [28], and ATACseq [29], which in concert with transcriptomes will provide a more complete understanding of astrocyte aging in the future.

Current datasets focusing on or including aged astrocytes deal with the above problems with variable strategies, including bulk sequencing of isolated/purified astrocytes, sc/snRNAseq of whole brain, dissected regions, or purified cells, spatial transcriptomics, and integrative/ multiomic strategies (Table 1). Most characterize mice as a model for mammalian aging; however, high quality data across the model organism spectrum provide common cellular aging insights across species. Existing knowledge of aging genetics in the worm (*Caenorhabditis elegans*) and the fruit fly (*Drosophila melanogaster*) make these systems compelling models for study of astrocytes and astrocyte-like cells during aging, though size and dissociation constraints have thus far limited sequencing strategies mostly to bulk or whole-animal sc/snRNAseq. As astrocytes/astrocyte-like cells become increasingly well defined in these organisms, transcriptional identification especially in established lifespan-altering mutants and manipulations will prove valuable. From the non-model organism perspective, human postmortem brain aging sequencing efforts are certainly the most relevant to human aging but suffer from limited availability of high-quality tissue. Existing aged human brain samples are often used as age-matched controls for disease studies, omitting a key comparison to young, healthy samples. The inclusion of young controls even in disease-targeted studies could help identify astrocyte phenotypes of normal aging, versus those unique to pathologies.

**Table 1:** Currently available astrocyte aged versus young sequencing datasets

Species	Strategy	Region	Ages	Notes	Reference
*C. elegans*	ScRNAseq (10 × v2)	Whole worm	1 d, 3 d, 5 d, 8 d, 11 d, 15 d	Cephalic sheath glia most resemble vertebrate astrocytes, *gon-2(q388ts)* mutants additionally sorted for somatic cells	[30]
*C. elegans*	snRNAseq (10 × v2/v3)	Whole worm	1 d, 6 d, 12 d, 14 d	Cephalic sheath glia most resemble vertebrate astrocytes, N2 wild-type strain, includes comparison to lifespan-altering mutants	[31]
*Drosophila*	ScRNAseq (10 × v3.1)	Whole brain	5 d, 30 d, 50 d, 70 d	Glial cells contain several astrocyte-like glia Both males and females	[32]
Human	snRNAseq (10 × v3.1)	Prefrontal cortex	26–84 yr	Wide age range of male and female, control and psychiatric disease postmortem patient samples, including 33 controls ages 32-82	[33]
Human	snRNAseq (10X v3, 3.1)	Cortex (prefrontal)	0.4–0.6 yr (infant), 15–57 yr (adult), 82–104 yr (elderly)	~ 20,000 nuclei total per donor	[34]
Human	snSPLiTseq	Hippocampus	0–1 yr (infant), 2–6 yr (child), 13–18 yr (adolescent), 27–21 (adult), 85–95 (aging)	Both males and females at all ages. 27,525 astrocytes, approximately 2,500 aged astrocytes	[35]
Human	snRNAseq (10X v 3.1)	Dorsolateral prefrontal cortex	22–97 yr	Includes comparison to schizophrenia-affected individuals (97 unaffected, 94 with schizophrenia), approximately 180,000 astrocytes total	[36]
Mouse	*Gfap*-Cre; Rpl22-HA^fl/fl^ bulk RNAseq	Cortex (visual, motor, somatosensory), hypothalamus, cerebellum	4 mo, 2 yr	Astrocyte ribosomal pull-down, males only	[18]
Mouse	*Aldh1l1*-eGFP-L10a bulk RNAseq	Striatum, hippocampus, cortex	P7, P32, 10 w, 9.5 mo, 2 yr	Astrocyte ribosomal pull-down, includes LPS-induced inflammation, includes males and females	[37]
Mouse	scRNAseq (10 × v2)	Whole forebrain	2–3 mo, 21–23 mo	6,931 astrocytes (3,398 young, 3,533 old), males only	[38]
Mouse	Bulk RNAseq	Cortex (motor, visual, entorhinal), hippocampus (anterior, posterior), hypothalamus, thalamus, caudate, pons, medulla, cerebellum, olfactory bulb, corpus callosum, subventricular zone, choroid plexus	3 mo, 12 mo, 15 mo, 18 mo, 21 mo, 26 mo, 28 mo	Bulk RNAseq of whole tissue, includes males and females	[25]
	spatial transcriptomics (10 × Visium)	Whole hemisphere sections	6 mo, 18 mo, 21 mo	Males only	
	snRNAseq (10 × v3.1)	Anterior hippocampus, caudate	3 mo, 21 mo	Small output of astrocytes, includes males and females, hippocampus includes dietary restriction in females	
Mouse	scRNAseq (10 × v2)	Cerebellum, cortex, hippocampus, striatum	1 mo, 3 mo, 18 mo, 21 mo, 24 mo, 30 mo	592 astrocytes (454 3 month, 107 18 month, 31 24 month), includes males and females	[39]
Mouse	ScRNAseq (10 × v3)	Whole brain	2 mo, 18 mo	261,959 astrocytes plus ependymal cells and tanycytes, includes males and females, includes enriched samples sorted by cell type	[1]
Mouse	EasySci-RNAseq/ ATAC-seq	Whole brain	3 mo, 6 mo, 21 mo	Includes males and females	[40]
Mouse	*Aldh1l1*-RPL10a^eGFP^ Bulk RNAseq	Whole cortex	6–7 mo, 21–23 mo	Astrocyte ribosomal pull-down, includes circadian timing comparison within age, includes males and females	[41]
Mouse	snRNAseq (10X v3.1)	Cortex (frontal), striatum	1 mo, 21 mo	6,771 astrocytes (3,441 juvenile, 2,780 aged), females only	[42]
	MERFISH spatial transcriptomics	Brain sections	1 mo, 6 mo, 21 mo	37,480 astrocytes (16,297 21 mo, 8,352 6mo, 12,831 1mo), females only, includes LPS-induced inflammation	
Mouse	MERFISH	Forebrain (including olfactory bulb), cerebellum, brainstem	3mo—34.5mo	Both coronal (*n* = 20) and sagittal (*n* = 6) sections Includes exercise and rejuvenation manipulation experiments	[43]
Mouse, Marmoset	snRNAseq (10X v3.1)	Cortex (prefrontal, motor), striatum, thalamus	Marmoset: embryo, neonate, 7 mo, 14 mo, 30 mo, 11–13 yr Mouse: E18.5, P4, P14, P32, P90, 90 w	~ 170,000 astrocytes across both species and all ages	[44]

A non-exhaustive list of aging astrocyte transcriptomic datasets

*Abbreviations*: *d* days, *E* Embryonic day, *gw* Gestational weeks, *mo* Month, *P* Postnatal day, *RNAseq* RNA sequencing, *sc/snRNAseq* Single cell/nucleus RNA sequencing, *w* Weeks, *yr* Year

Despite challenges in sequencing astrocytes and other cells from the aged brain [45, 46], the existing body of literature highlights several areas of interest that may be altered in normal aging, which we discuss below.

### Regional heterogeneity of astrocyte aging

Though astrocyte regional heterogeneity is increasingly well-defined in young animals (e.g. [23]), it is not well understood how it contributes to brain aging. Subregion-specific bulk astrocyte RNA sequencing demonstrates that astrocytes across brain regions display different aging timelines, with regions such as hippocampus, hypothalamus, and cerebellum beginning to display an aging signature earlier in adulthood than in cortex [18, 25, 37]. We thus recommend avoiding generalization of age-related gene expression changes from one CNS region to another, as changes in each region can diverge – particularly across the decades-long lifespans of species like human. Notably, the speed of transcriptional aging by region does not exactly recapitulate regional vulnerability observed in aging-related degenerative disease. These data suggest an open question in interpretation of aged transcriptomes: do observed changes represent protective adaptations, pathogenic changes, or are they unrelated to the generation of aging-related disease?

Though regionally variable, aged astrocytes seem to generally upregulate genes associated with white matter astrocyte identity at younger ages [25]. Single cell resolution spatial transcriptomics suggests that these age-related changes are correlated with proximity to oligodendrocytes [42]. In young adult tissues, white matter astrocytes are strong responders to insults like peripheral lipopolysaccharide (LPS) injection, suggesting that aging-related white matter-like gene expression may represent astrocyte integration of insults to oligodendrocytes and myelin-dense tissue occurring across normal aging [24]. Similar signatures are also observed at the brain borders, suggesting astrocytes in contact with other tissues such as the meninges and the blood–brain barrier may encounter and respond to more damage cues [42].

Regional heterogeneity among aging astrocytes suggests that astrocyte cell–cell interactions and exposure to unique insults may be among the most important factors in determining aging-dependent transcriptional changes [43]. To understand how astrocytes change in aging, we must disentangle how proximity to blood vessels or high-density myelin in the case of the white matter, for example, may impact eventual phenotypes. Differences observed in regional transcriptomes suggest that aging-associated astrocyte states may represent a summation of variable insults occurring across the lifetime. If such insults fail to resolve, they may leave astrocytes reacting to states usually present only in inflammation or damage in young animals.

### What is the role of astrocyte reactivity in normal aging?

Astrocyte reactivity is multifaceted, region and context specific, and variable across organisms. Transcriptional and protein-level studies have demonstrated an increase specifically in inflammation-induced neurotoxic reactivity in aging [37], suggesting that astrocyte toxicity has the potential to underly age-related neuronal loss. Astrocyte reactivity in neuroinflammation in vivo is heterogenous, however, and phenotypes of newly identified inflammatory reactive substates under stress and aging are unknown [24]. It is unclear which substates of astrocyte reactivity may drive aging-related dysfunction and disease and which may be required for recovery from damage, as study of even the best functionally defined substates has most frequently been performed under severe or terminal injury/infection models, such as amyotrophic lateral sclerosis models, optic nerve crush, or sepsis [10–12].

A particular reactive astrocyte substate may be detrimental in one context but have protective roles in another, a possibility that is under-investigated in aging. For example, it is unknown whether neurotoxic reactive astrocytes may have adaptive roles in resolving damage, by culling damaged or infected neurons before an area of injury expands or infection propagates throughout the CNS. Such is the case following prion infection in mice, where global knockout of neurotoxic astrocyte inducing cytokines accelerates disease [47]. Neurotoxic reactivity may also play a role in development or re-growth, as C3 + putatively neurotoxic astrocytes are associated with stem cell niches in the human embryonic brain [48]. Similarly, scar-forming astrocytes in physical or ischemic CNS injuries participate in fibrosis but can help to promote regrowth of neurons across lesions and limit expansion of inflammation and immune infiltration that could further harm unaffected tissue [49–52]. Thus, astrocyte reactivity may enact divergent outcomes depending on context. In microglia, for example, identical substates have both beneficial and deleterious roles in the contexts of development and disease [53, 54]. As sequencing expands our understanding of reactive astrocyte substates, more hypothesis-driven work is necessary to understand the interplay in protection and pathology.

Examination of reactivity in normal aging has primarily focused on functionally defined substates, most notably neurotoxic reactive astrocytes [37]. However, more reactive astrocyte states are increasingly defined that can now be explored in normal aging [24]. Notably, risk of stroke and other blood–brain barrier disruptions increases with age [55, 56], raising the possibility that astrocytes reacting to these conditions may increase across the lifespan. More work is needed to understand how insults known to produce unique astrocyte phenotypes in young animals impact astrocytic responses in old animals, particularly from the lens of functional outcomes. Insults to proteostasis, energy generation, and damage degradation have known aging-related effects in many tissues but are understudied in astrocytes. Reactivity to these aging-related insults remains an interesting unexplored area.

### Immune responses and immune-astrocyte interactions in age

As organisms accumulate insults and damage across the lifespan, they also upregulate immune responses in a phenotype known as “inflammaging” [for review see 57]. Astrocytes are strongly environmentally responsive, sensing inflammatory cytokines and altering their transcriptional profiles and functions accordingly [15]. Indeed, immune related genes, predominantly those in the complement pathway, are among the most upregulated and well-validated astrocytic age-related changes in gene expression. The complement factor *C4b* is among the most reproducibly increased in aged astrocytes [18, 25, 37, 38]. Though the role of the complement cascade in synaptic pruning is best described in microglia, recent work suggests a potential role for *C4b* in astrocyte-mediated debris engulfment [58]. However, *C3*, another complement factor broadly associated with inflammatory neurotoxic astrocyte reactivity, is also increased in normally aged astrocytes but associated with decreased phagocytic function in vitro [37]. These data suggest that astrocytes may alter their phagocytic capacity bidirectionally in aging, which could lead to altered specificity or compensatory activity in pruning and cell engulfment.

Astrocytes from aged brains of rodents and humans consistently exhibit upregulation of several cytokines and chemokines, perhaps unsurprisingly as the immune environment is known to become more pro-inflammatory with age [18, 25, 37, 57]. Astrocytes in the adult brain can adopt an interferon (IFN)-responsive phenotype following peripheral infection signals such as those derived from LPS injection and disease states like Alzheimer’s disease (AD), in which IFN-stimulated genes (ISGs) such as *Cxcl10* are upregulated [22, 24, 25, 37, 59]. This IFN-responsive reactive astrocyte (IRRA) state has also been described in human postmortem brain samples from patients with multiple sclerosis [60] and AD [61, 62] and in mouse demyelinating and AD models [62, 63]. A subset of these ISGs is also upregulated in normally aged astrocytes, suggesting that IRRAS may represent a long-term infection-responsive phenotype that perhaps fails to successfully recover as would occur in young animals [24, 25]. Induction of ISGs may alter the brain microenvironment by changing peripheral immune cell recruitment into the parenchyma by secretion of chemokines like CXCL10 and CCL2 [64–67], raising the possibility that aged astrocytes could differ from their younger counterparts in their ability to recruit central or peripheral immune cells to sites of pathology. Astrocytes are powerfully responsive to extrinsic input from immune cells, suggesting that these and other immune-related changes could occur due to increasing organismal inflammatory cytokines and damage signals. Microglia, for example, increase their responses to inflammatory challenge with age, expressing and secreting more cytokines that influence astrocytic function, such as TNF and IFNs [68].

Existing characterizations of transcriptional changes associated with astrocyte reactivity include upregulation of several Serpin family member genes, so named for their roles as serine protease inhibitors [15]. Astrocyte datasets demonstrate Serpin-family transcriptional upregulation with age, though the specific identities of these factors differ across studies [18, 37]. The roles of Serpins extend beyond protease regulation alone, and have consequences for inflammation and protein homeostasis, key features of normal and damage-responsive astrocyte functions (for review see [69]). Few studies have directly interrogated the roles of these proteins in astrocytes, though they may impact cytokine levels in the hippocampus and cortex and neuronal health [70].

### Astrocytes in senescence

Although the CNS is composed primarily of post-mitotic cells [71, 72], DNA damage and cellular senescence nonetheless impact cellular health in the brain. Senescence in the periphery implicates aberrant irreversible cell cycle exit, as in the case of replicative senescence; however, senescence in the CNS benefits from a more expansive definition, incorporating markers of DNA damage and downstream metabolic dysfunction (for review see [73, 74]). Astrocyte/astrocyte-like glial senescence has traditionally been best described in the *Drosophila* model system, in which they upregulate senescence-associated markers, such as the transcription factor AP1 and increased β-galactosidase activity, during normal aging [75, 76]. Recent work suggests that lipid accumulating “senescent” astrocyte-like cells in Drosophila can induce this senescence-associated phenotype in other astrocyte-like cells, and that inhibiting these signals can improve organismal health across the lifespan [75]. Astrocytes are also key regulators of brain permeability and respond to blood and CSF-derived peripheral cues in vertebrates; thus, response to DNA or mitochondrial damage and cellular senescence signaling could also be accomplished via responsiveness to DNA damage and irreversible cell cycle exit in peripheral cells. Further investigation is required to comprehensively understand how senescence impacts astrocyte aging in mammals.

### Loss of astrocyte synaptic support in aging

Astrocytes enable synaptic function and neuronal activity by physically enmeshing neurons in extracellular matrix components, by supplying neurotrophic and synaptogenic materials, and by transporting membrane components like cholesterol [77–80]. As animals age, synaptic stability and dendritic spine number decrease, suggesting an overall failure of neuronal support functions [5] (for review see [6, 7]). Aging astrocytes likely contribute, as they upregulate negative synaptic regulator *Sparc*, while generally downregulating the synaptogenic thrombospondin-family genes, though other positive regulators like *Sparcl1* are upregulated, complicating the interpretation of these data [18, 37]. Neurotoxic reactive astrocytes, which are increasingly abundant with normal age [37], also have impaired synaptic support functions [15]. Other types of reactive astrocytes, like those responsive to ischemia and injury, have notable roles in synapse regulation in young adult animals, but these whether these functional changes also occur in aging remains largely unknown [16, 50, 81]. As risks of brain ischemia and other insults increase with age in humans, more work is necessary to fully interrogate how uniquely reactive substates impact neuronal function across aging. Together, these data suggest both a failure of aged astrocytes to stabilize synapses and a dysregulation in downstream elimination of the immature or damaged processes by both microglia and astrocytes.

### Decline of protein homeostasis in aged astrocytes

To manage age-related insults and damage, organisms must appropriately address damaged organelles, misfolded proteins, and other cellular damage. Maintaining proteostasis is a key feature of healthy aging, and existing data surrounding unfolding protein responses suggest that astrocytes manage the majority of this signaling in the brain across phyla [82–84]. Astrocytes highly express several chaperones and protein homeostasis regulators, including the HSP20 gene *Hspb1* and the extracellular chaperone/ lipid transport APOJ apolipoprotein gene *Clu*, suggesting important roles for astrocytes in mediating proteostasis, especially under stressors like sepsis-related inflammation [24]. In one study, proteostasis genes were three (*Hsph1*, *Dnajb1*, and *Ahsa1*) out of only seven shared down-regulated genes across regions of the aged brain, both emphasizing the subtlety and heterogeneity in astrocyte aging across regions and proposing a general dysfunction of proteostasis may occur across aged astrocytes [25]. Existence of a subset of aging-associated astrocytes in the 2-year-old mouse hippocampus that display impairments in protein degradation and homeostasis also corroborates this hypothesis [85]. One study also identified several HSP70 cytosolic chaperones as among the most downregulated with age [18]. Astrocytes have been previously shown to direct protein misfolding responses across tissues in *C. elegans* and mice [82–84, 86]. As protein misfolding stress responses are enacted by transcriptional responses, the proximity of astrocyte somata to neuronal distal processes enables astrocytes to better protect neurons and other cells from damage under stress by these responses. Failure of these pathways during normal aging may sensitize the brain to diseases of protein misfolding with age, a notable phenotype of age-related neurodegenerative diseases.

### Aged astrocyte lipid dysbiosis

**Fig. 2:**
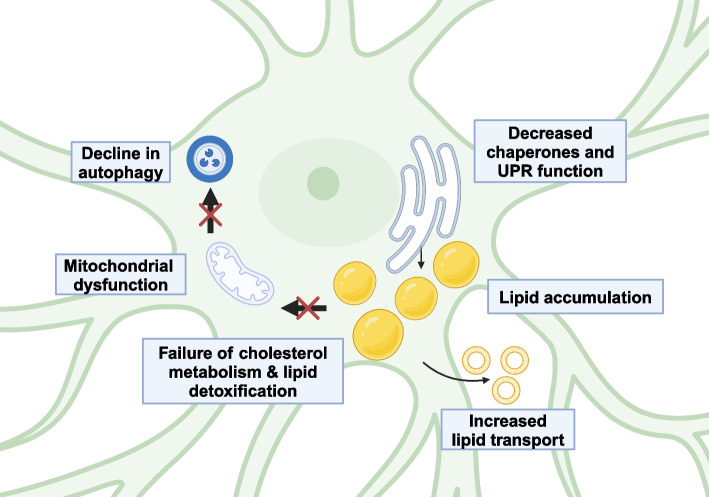
Aging-related changes in astrocyte cellular functions. A decline in proteostasis and chaperone function may lead to organelle stress. Lipid droplets, which derive from the endoplasmic reticulum, accumulate in age, associated with downregulation of lipid metabolism transcripts and upregulation of lipid transport transcripts. Accompanied with aging-related decline in mitochondrial function and failure of autophagy, this may lead to accumulation of potentially toxic lipid species. Figure made using Biorender.com

Changes in lipid-associated gene expression, particularly of factors involved in cholesterol metabolism and transport, are also common transcriptional changes in astrocyte aging [18]. Among these factors are genes that regulate cholesterol synthesis, which are primarily downregulated, and several lipid transporters, largely upregulated [18]. Homeostatic astrocytes supply neurons and microglia with cholesterol to mediate membrane health and neuronal activity. Thus, this shift, which couples the downregulation of metabolic enzymes with increased transport capacity, may be the cell’s attempt to compensate for the loss of valuable products [77, 87]. Previous work in microglia suggests lipid metabolic imbalances causing accumulation with age are correlated with altered phagocytic capacity, which is not well understood in astrocytes but could lead to similar phenotypes [88, 89]. If indeed aged astrocytes are plagued by general metabolic dysfunction, this may have disastrous consequences for neural activity and nutrient transport between the cell types (Fig. 2).

Metabolism of lipids is highly segregated in the CNS, as astrocytes serve a central role in accumulation and detoxification of lipid species under stress. Stroke, hyperactivity/glutamate excitotoxicity, neuronal reactive oxygen species, and peripheral metabolic disfunction cause astrocytes to accumulate lipid droplets, neutral lipid storage organelles made up primarily of triacylglycerols (TAGs) and sterol esters [9, 90, 91]. Neurons offload potentially damaging lipid species, including peroxidated lipids, to astrocytes, which neutralize and store them until they can be degraded [9]. In addition to lipid-mediated neuroprotection, however, astrocytes enact neurotoxicity via secretion of saturated long chain fatty acid lipids that kill sensitive and/or damaged neurons [92]. Thus, the astrocytic balance opposing lipid accumulation with elongation and secretion is a determinant of neuronal survival under stress.

Aging is a key modulator of astrocytic lipid homeostasis across phyla, and astrocytes accumulate lipid droplets in normal aging [18, 75, 93, 94]. In the 5xFAD mouse model of AD, astrocytes exhibit failure of lipid metabolism and increased lipid accumulation, suggesting these imbalances may be disease-associated in vivo [95]. The balance between lipid storage versus release from lipid droplet breakdown is mediated by signals known to change across the lifespan, such as insulin and inflammatory cytokines, and can be influenced by senescence [75]. The consequences of lipid dysbiosis in astrocytes are not fully understood in the context of normal aging; however, these data suggest a context in which aged astrocytes may lose key homeostatic functions and gain potential for toxicity.

### Mitochondrial dysfunction in aged astrocytes

Mitochondrial damage increases as organisms age and the ability to resolve such damage via appropriate stress responses decreases. This imbalance is correlated with failure of mitophagy (the autophagy-mediated clearance of damaged mitochondria), decreased energy production to support cell functions, and altered organellar phenotypes such as mitochondrial membrane potential and fusion/fission [for review see 4].

Damaged mitochondria derived from other cells can be taken up by astrocytes, and conversely healthy astrocyte-derived mitochondria can be found in neurons under stress [96, 97], suggesting that astrocytes maintain neuronal mitochondrial quality under stressful conditions. In general, mitochondrial quality control declines with age, including the degradation of damaged mitochondria by mitophagy [for review see 4]. Astrocytic mitophagy, particularly on behalf of neurons, is thus central to healthy aging in the nervous system.

Although energy production in astrocytes is biased towards glycolysis, astrocyte mitochondrial functions remain key to CNS health across the lifespan. In human tissue, Ramen spectra of aged astrocytes suggest that electron transport chain function declines, associated with age-related accumulation of lipids that may perturb general brain energy production [98]. Astrocyte-specific perturbation of mitochondrial health via conditional deletion of the transcription factor *Tfam* results in neurodegeneration and cognitive dysfunction, suggesting that health of astrocytic mitochondria is integral for brain-wide functions, and perturbing astrocyte mitochondria leads to lipid dysbiosis and immune signaling as in phenotypes described above [95]. If mitochondrial health in astrocytes is lost or perturbed in normal aging as in other cells, this loss of function could explain sensitivity to aging-related neurodegeneration.

### Changes in neurodegenerative disease genetic risk factors across age in astrocytes

Though neurons are principally targeted by cell death/loss in aging-related neurodegenerative diseases and dementias such as AD and Parkinson’s Disease (PD), loci associated with genetic risk for these diseases are frequently associated with gene expression in non-neuronal cells, and some are highly expressed in astrocytes (for review see [99]). Though the functional consequences of aging-related changes in these transcripts are poorly described, further study of these changes may provide insight to neurodegenerative disease pathogenesis.

Loci associated with AD risk in genome-wide association studies include genes encoding two astrocytic apolipoproteins – APOE (gene name *Apoe*) and APOJ (also known as Clusterin; gene name *Clu*) [100]. Interestingly *Apoe* is highly enriched, and *Clu* nearly exclusively expressed in astrocytes within the brain [101, 102]. Apolipoproteins canonically transport lipid species between cells, though APOJ also aids in protein homeostasis as described above, and both APOE and APOJ are associated with astrocyte lipotoxicity [92]. However, neither *Apoe* nor *Clu* are significantly differentially expressed in mouse astrocytes in aging in AD-sensitive regions like the hippocampus or cortex [18, 37]; nor are differences in APOE/APOJ detectable at the protein level [103]. It remains unknown how differences in splicing or post-translational modifications might change across the lifetime, especially in astrocytes. As both proteins are implicated in astrocyte-mediated lipotoxicity, these data may suggest a role for this pathway in AD pathogenesis that may occur as inflammation increases across the lifespan.

Though PD-associated risk genes overall are not exclusively expressed in astrocytes, there are likely astrocyte roles for some risk genes that have yet to be explored in normal aging (for review see [104]). Rare mutations in the PINK1-parkin pathway led to an association between mitochondrial dysfunction, failure of mitophagy, and PD pathogenesis [104]. As described above, organelle quality control and specifically mitophagy decline with age [4], suggesting decline in mitochondrial function might be responsible for age-related risk for PD. Multiple PINK1-parkin pathway genes are expressed in human astrocytes at comparable or higher levels than in neurons [102] and PINK1 itself may be more ubiquitinated in mouse astrocytes than other cells [105]. There is some evidence *Pink1* transcripts increase in aged striatal astrocytes [37]; however, as PINK1 is constitutively targeted for degradation at baseline and only stabilized under stress, the impact of age-related transcriptional upregulation is ambiguous.

In sum, though genetic risk factors for aging-related neurological diseases more often associated with gene expression in non-neuronal cells, including astrocytes, little is known about how these factors change functionally in these cells across the course of normal aging. Examining relevant pathways in aged astrocytes will continue to highlight important components of neurodegenerative disease pathogenesis.

## Conclusions

Despite major efforts to characterize whole brain changes in aging in non-neuronal cells, research has observed fewer consistent transcriptional changes in aged astrocytes than we would perhaps expect based on differences in phenotype and stress tolerance in the old brain. Even within well-controlled datasets individual variability is high, and the specific ages and brain regions collected of samples within the categories of “old” and “young” have major effects on dataset outputs. This variability likely masks important heterogeneous yet subtle biological changes that affect astrocytic aging. In the next phase of astrocyte aging research, we will need to revisit, integrate, and reanalyze these data in the context of heterogeneity that is now common in our understanding of astrocyte biology [24]. New tools that combine transcriptional phenotypes with metabolic, protein level, and functional readouts throughout the brain at single-cell resolution will more faithfully represent aging-related changes in these cells. However, existing data suggest that astrocytes, especially aged astrocytes, remain strongly transcriptionally responsive to local cell–cell signaling, peripheral insults, and whole-organism state changes. Just as “astrocyte reactivity” is too general to describe each uniquely responsive phenotype of astrocytes experiencing stress and damage, astrocyte aging is a multifaceted system of changes best detectable with single cell resolution datasets across regions and modalities.

**Fig. 3:**
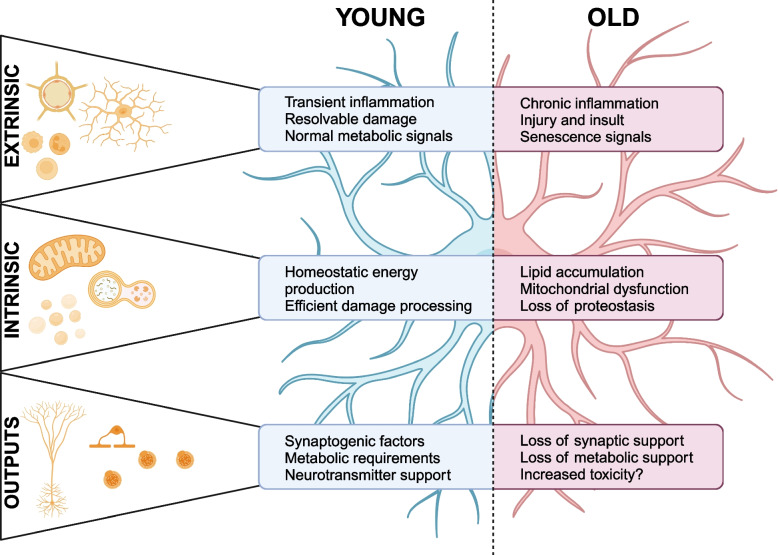
Changes in cellular environments, including changing damage/inflammation signals and metabolic dysfunction, contribute to altered intracellular functions and maintenance of homeostasis in astrocytes. In turn, these dysfunctions are associated with worsened outcomes for neurons and other CNS cells, astrocytes lose their ability to provide supportive factors and potentially gain toxic functions. Figure made using Biorender.com

Astrocyte research has arrived at a key question in aging: do aging phenotypes represent the natural extension of homeostatic conditions, or is aging itself an insult to the system? Should we think of chronological age in the brain as disease or simply continuation? The subtle changes currently observed in study of aged astrocytes suggest that the cells are largely predisposed by their environments and basal states together to become poor assistants of neurons under stress, and at worst, contribute to neuronal death. Astrocytes lose key functions required for neuronal support with age, failing to process damage, support synapse growth, and maintain energy homeostasis. Meanwhile, they increasingly experience immune and senescent challenges that bias towards harmful gain-of-function states such as inhibition of neuronal growth or neurotoxicity (Fig. 3).

Aging is one of the most significant risk factors for the development of neurodegenerative disease [106]. The similarities of astrocyte aging-related changes and disease mechanisms (synapse loss, neurodegeneration, increased inflammation, and failure of damage processing) suggest a major role for astrocytes in aging-related predisposition to disease. Further, if indeed astrocyte neurotoxicity via increased inflammation is responsible for cell loss in neurodegeneration, then potential interventions against astrocyte aging may prove useful throughout normal aging for disease prevention.

## Abbreviations

AD: Alzheimer’s Disease
CNS: Central nervous system
CSF: Cerebrospinal fluid
DNA: Deoxyribonucleic acid
RNA: Ribonucleic acid
sc/snRNAseq: Single cell/nucleus RNA sequencing
IFN: Interferon
IRRA: Interferon responsive reactive astrocyte
ISG: Interferon response genes
LPS: Lipopolysaccharide
PD: Parkinson’s Disease
TAGs: Triacylglycerols
